# Mediators of Intervention Effects on Depressive Symptoms Among People Living With HIV: Secondary Analysis of a Mobile Health Randomized Controlled Trial Using Latent Growth Curve Modeling

**DOI:** 10.2196/15489

**Published:** 2019-11-15

**Authors:** Mengting Zhu, Weiping Cai, Linghua Li, Yan Guo, Aliza Monroe-Wise, Yiran Li, Chengbo Zeng, Jiaying Qiao, Zhimeng Xu, Hanxi Zhang, Yu Zeng, Cong Liu

**Affiliations:** 1 Department of Medical Statistics and Epidemiology, School of Public Health Sun Yat-sen University Guangzhou China; 2 Department of Infectious Diseases, Guangzhou Eighth People's Hospital Guangzhou China; 3 Center for Migrant Health Policy Sun Yat-sen University Guangzhou China; 4 Sun Yat-sen Global Health Institute Sun Yat-sen University Guangzhou China; 5 Department of Global Health University of Washington Seattle, WA United States; 6 South Carolina SmartState Center of Healthcare Quality, Arnold School of Public Health University of South Carolina Columbia, SC United States; 7 Department of Health Promotion, Education, and Behavior, Arnold School of Public Health University of South Carolina Columbia, SC United States; 8 National Center of AIDS/STD Control and Prevention China Center for Disease Control Beijing China

**Keywords:** mobile health, depression, HIV, randomized controlled trial, longitudinal studies

## Abstract

**Background:**

Although several studies have investigated the effects of mobile health (mHealth) interventions on depression among people living with HIV, few studies have explored mediators of mHealth-based interventions to improve mental health in people living with HIV. Identifying influential mediators may enhance and refine effective components of mHealth interventions to improve mental health of people living with HIV.

**Objective:**

This study aimed to examine mediating factors of the effects of a mHealth intervention, *Run4Love*, designed to reduce depression among people living with HIV using 4 time-point measurement data.

**Methods:**

This study used data from a randomized controlled trial of a mHealth intervention among people living with HIV with elevated depressive symptoms in Guangzhou, China. A total of 300 patients were assigned to receive either the mHealth intervention (n=150) or a waitlist control group (n=150) through computer-generated block randomization. Depressive symptoms, coping, and HIV-related stigma were measured at baseline, 3-, 6-, and 9-month follow-ups. The latent growth curve model was used to examine the effects of the intervention on depressive symptoms via potential mediators. Mediating effects were estimated using bias-corrected 95% bootstrapped CIs (BCIs) with resampling of 5000.

**Results:**

Enhanced positive coping and reduced HIV-related stigma served as effective treatment mediators in the mHealth intervention. Specially, there was a significant indirect effect of the mHealth intervention on the slope of depressive symptoms via the slope of positive coping (beta=–2.86; 95% BCI –4.78 to –0.94). The indirect effect of the mHealth intervention on the slope of depressive symptoms via the slope of HIV-related stigma was also statistically significant (beta=–1.71; 95% BCI –3.03 to –0.40). These findings indicated that enhancement of positive coping and reduction of HIV-related stigma were important mediating factors of the mHealth intervention in reducing depression among people living with HIV.

**Conclusions:**

This study revealed the underlying mediators of a mHealth intervention to reduce depression among people living with HIV using latent growth curve model and 4 time-point longitudinal measurement data. The study results underscored the importance of improving positive coping skills and mitigating HIV-related stigma in mHealth interventions to reduce depression among people living with HIV.

## Introduction

### Background

Depression is highly prevalent among people living with HIV (PLWH) [1]. Data from a systematic review in China indicate that the pooled prevalence of depressive symptoms is 50.8% in PLWH [2]. In contrast, the prevalence of depressive symptoms in the general population is 17.1% [3]. Depression is consistently associated with impaired role functioning, worsened antiretroviral therapy adherence, elevated risks of HIV-related morbidity and mortality, and increased health care costs [4-6]. Although depression is highly prevalent and disabling, it remains greatly undertreated worldwide with more than 90% of those diagnosed with depression in China and India not on treatment and more than 50% untreated even in many high-resource settings [7,8].

Literature has shown that interventions such as cognitive behavioral stress management (CBSM) are effective at reducing depression in various populations including PLWH [9-11]. However, few studies have explored mediators of how such interventions reduce depression. Mediators are statistical constructs that constitute 1 type of mechanism for how an intervention affects outcomes [12,13]. Understanding influential mediators is critical for understanding how interventions may achieve effective outcomes and help enhance and refine intervention components for future implementation or scaling up to improve mental health of PLWH [14].

Previous studies have indicated that factors such as coping and stigma may be strong predictors of depression. Coping strategies have been defined as individuals’ emotional, cognitive, and behavioral attempts in response to stressful events [15,16]. HIV-related stigma is defined as processes that devalue, label, negatively stereotype, or treat unfairly objects or people associated with HIV [17,18]. Higher positive coping was associated with lower depressive symptoms [19]. Higher levels of HIV-related stigma and negative coping were associated with higher depressive symptoms [19,20]. CBSM can instruct patients to modify maladaptive cognitions such as HIV-related stigma, acquire social support, and engage in more adaptive behaviors such as positive coping to reduce depression and facilitate adjustment [21,22]. In the Stigma and HIV Disparities Model proposed by Earnshaw, enhancing positive coping could improve resilience to stigma and ultimately improve health outcomes among PLWH [23]. Existing studies have suggested that coping and stigma might be important factors in influencing the effects of psychological treatments on depression among PLWH [19,24,25]. For example, Tshabalala and colleagues observed that HIV-positive women randomized to cognitive behavioral intervention group reported greater reduction in depression than the control group. In addition, a significant reduction in HIV-related stigma and negative coping and improvement in positive coping were observed in comparison with the control group [25]. Utilizing qualitative analysis, the same study found that reasons for the effectiveness of the cognitive behavioral intervention to reduce depression might be the enhancement of participants’ coping and assertiveness skills and reduction in HIV-related stigma during the intervention. Similarly, another trial among HIV-seropositive men who have sex with men in the United States also reported that CBSM intervention was efficacious in improving cognitive coping strategies and improved coping might be an important determinant of both depression and anxiety reduction [21].

However, the few studies that explored intervention mediators of depression reduction were mostly conducted within interventions that occurred in clinic settings and were delivered face-to-face. To the best of the knowledge of the authors, no study has been done based on mobile health (mHealth) interventions [21,25]. It is not clear that factors effective in mediating intervention effects on depression reduction in traditional delivery settings remain effective in mHealth interventions. With the wide coverage of smart mobile phones and emerging literature on the initial effectiveness of mHealth interventions, it is important to understand the underlying mechanisms and mediating factors for mHealth interventions, especially in comparison with traditional face-to-face interventions [26-29]. As mHealth interventions have the potential to reach a large population with less stigma, lower cost, and increased convenience, more research is needed for greater understanding of mediators of effective mHealth interventions [30-33].

In addition, existing studies mostly used qualitative analysis or pre-post measurements to examine mediators of interventions for depression reduction; longitudinal studies with multiple waves are lacking [21,25]. As pre and post measurements contain minimal information on individual changes, longitudinal data with multiple (≥3) waves allow investigation on how factors change and how these changes are related to health outcomes over time [12,34]. Mediators should temporally precede the outcomes to demonstrate causal temporal relationships [12,13].

### Objectives

To bridge the gap in the existing literature, this study aimed to examine mediating factors of the effects of an mHealth intervention, Run4Love, designed for depression reduction among PLWH based on CBSM with 4 time-point measurement data. We hypothesized that both coping and HIV-related stigma would play important roles in mediating the effects of the Run4Love mHealth intervention. Specifically, we hypothesized that the intervention would improve positive coping and decrease HIV-related stigma, which in turn would lead to reduced depression among PLWH.

## Methods

### Design and Procedure

This study used data from a randomized controlled trial (RCT) of an mHealth intervention, Run4Love, for depression reduction among PLWH in China. This study is a secondary analysis of the Run4Love RCT (ChiCTR-IPR-17012606) [35]. Participants were recruited by trained research staff at the outpatient clinic of a large hospital designated for HIV treatment. The study was conducted from September 9, 2017, to October 1, 2018, at Guangzhou Eighth People's Hospital in Guangzhou, China. Details of study design and procedures can be found in the study protocol [35]. Briefly, patients who met the eligibility criteria were provided with a study pamphlet that described research procedures and were invited to join the study. A total of 300 patients were enrolled in the study. Following enrollment, the patients were assigned to either the Run4Love mHealth intervention (n=150) or the waitlist control group (n=150) through computer-generated block randomization with a block size of 4 and an allocation ratio of 1:1. By the nature of the trial design, neither research staff nor participants were blinded to the Run4Love mHealth intervention. The allocation sequence was not concealed from the research staff. The duration of the intervention was 3 months, and participants were followed up to 6 months after the intervention. Participants were assessed at baseline, 3-month follow-up, 6-month follow-up, and 9-month follow-up using electronic questionnaires on tablets. These assessments were conducted during face-to-face sessions with the (nonblinded) research staff at the outpatient clinic. The primary outcome was depressive symptoms assessed by the Center for Epidemiological Studies Depression Scale (CES-D) [36]. Written informed consent was obtained from each participant before data collection. Participants who completed each of the study assessments received 50 RMB (ie, about US $8) or gifts of equivalent value for completion of each survey. The Consolidated Standards of Reporting Trials EHEALTH checklist is shown in Multimedia Appendix 1.

### Participants

Patients were eligible to participate if they were aged 18 years or older, HIV seropositive, having elevated depressive symptoms (CES-D≥16), willing to provide hair samples, and using WeChat, the most popular application for instant communication in China [37]. Hair samples were collected to test the cortisol content as a biomarker of chronic stress. Patients were excluded if they were taking psychiatric drugs, unable to finish questionnaires because of mental or other illnesses or other reasons, unable to read or listen to the materials sent via WeChat (ie, short articles, audios, and posters), unable to engage in physical activities because of medical reasons, or had hair permed or dyed in the past 3 months. Those who refused to participate provided information on specific reasons for refusal. This study was approved by the institutional review board of Sun Yat-sen University in Guangzhou, China.

### Run4Love Mobile Health Intervention Program

Participants in the intervention group participated in a 3-month mHealth intervention delivered by the enhanced WeChat platform, consisting of the adapted CBSM course and regular physical activity promotion [37]. The adapted CBSM course consisted of 9 sessions and 3 review sessions on coping skills and stress reduction management such as practices of effective coping skills, cognitive distortions, meditation, and breathing. Sessions were in multiple formats, including audio clips, articles, and posters. On average, the articles were 1300 words and took about 5 min to read; the audios were 5 to 10 min. The physical activity promotion program included information about benefits of and guidance on regular exercise and a healthy diet. Participants in the intervention group received CBSM and physical activity promotion information on their WeChat account 3 to 5 times a week. Participants could review the materials they had received at any time.

The Run4Love intervention also included 5 phone calls from research staff at the first week and 1, 2, 5, and 8 months after enrollment. The purpose of the phone calls was to confirm participation and proper use of the platform at the first week and to offer social support, facilitate intervention implementation, identify barriers to adherence, and provide feedback on mental health in subsequent months. All calls had a script for reference. The calls lasted for an average of 10 min during the first week and 15 min during subsequent weeks. The intervention is described in detail elsewhere [35].

### Control Program

Participants in the control group received a brochure on nutrition and healthy living in addition to usual care for HIV treatment. Moreover, they were offered to receive the Run4Love intervention as soon as the study ended (ie, 9 months after enrollment).

### Measures

#### Depressive Symptoms

Depressive symptoms were assessed by the CES-D with good reliability and validity [36]. The 20-item measure included 4 subscales: depressed affect, positive affect, interpersonal relationship, and somatic and retarded activity. Higher scores indicated higher levels of depressive symptoms. All items used a 4-point Likert scale from 0 (“Rarely or none of the time”) to 3 (“Most or all of the time”). The total scores ranged from 0 to 60, with scores of 16 or above being considered as elevated depressive symptoms. The CES-D scale demonstrated good internal consistency, with Cronbach alphas ranging from .88 to .93 across the 4 waves of assessment.

#### Coping

The 20-item Simplified Ways of Coping Questionnaire (SWCQ) was used to assess coping with proven reliability and validity [38]. The SWCQ assessed different attitudes and measures of coping that people adopted in their daily lives using a 4-point Likert scale from 0 (“Not used at all”) to 3 (“Used frequently”). The SWCQ measurement consisted of 2 subscales: positive coping (12 items) and negative coping (8 items). The positive coping scores ranged from 0 to 36, with higher scores indicating better positive coping. Cronbach alphas for positive coping ranged from .85 to .93 across 4 time points. The negative coping scores ranged from 0 to 24. Higher scores indicated higher levels of negative coping. Cronbach alphas for negative coping ranged from .65 to .74 across 4 time points.

#### HIV-Related Stigma

HIV-related stigma was measured by 14 items derived from the HIV Stigma Scale with good reliability and validity among Chinese PLWH [39,40]. The measurement included 2 subscales: perceived stigma (6 items) and internalized stigma (8 items). Each item used a 4-point Likert scale from 1 (“strongly disagree”) to 4 (“strongly agree”). The total scores ranged from 14 to 56, with higher scores indicating higher levels of HIV-related stigma. An example of the items assessing perceived stigma was “People with HIV lose their jobs when their employers learn.” A sample item of internalized stigma was “I feel guilty because I have HIV.” The HIV-related stigma scale demonstrated good internal consistency, with Cronbach alphas ranging from .92 to .96 across the 4 waves of assessment.

### Demographic Variables

Demographic variables included age, gender, educational level, sexual orientation, marital status, employment status, family monthly income, and duration since HIV diagnosis.

### Statistical Analysis

All analyses were conducted based on intention-to-treat principle. Baseline characteristics were compared between the intervention and control groups using *t* tests or Wilcoxon rank-sum tests for numeric outcomes and chi-square tests for categorical variables. All statistical tests were 2-sided, and *P* value <.05 was considered statistically significant.

The latent growth curve model (LGCM) was used to examine mediating factors of the effects of the mHealth intervention on depressive symptoms among PLWH using 4 time-point measurement data. As an extension of structural equation modeling (SEM), LGCM allowed simultaneous analysis of multiple time points, thus potentially providing more accurate estimation of changes over time [41]. LGCM was suited in this study because it accommodated longitudinal data with multiple waves where both the mediator and the outcome changed simultaneously over time, therefore allowing researchers to address longitudinal mediation [42,43]. Though the framework of LGCM was similar to that of SEM, LGCM allowed for estimation of inter- and intraindividual variation over time and exploration of predictors of these individual differences [34,43]. Repeated measurements collected at 4 time points (ie, at baseline and 3-, 6-, and 9-month follow-ups) were included as observed indicators, with the latent intercept (ie, initial status) and slope (ie, rate of change) factors being estimated. With the longitudinal data, the parallel-process LGCM allowed exploration of the effects of the intervention on changes of variables of interest, or the growth rates (ie, slopes) of the variables of interest.

A stepwise approach was used to examine the mediating effects, which were widely used with longitudinal data [43]. First, unconditional parallel-process LGCM of HIV-related stigma, coping, and depressive symptoms were specified to estimate growth trajectories and each construct’s temporal stability for both groups. Factor loadings for the intercept at each time point were set to 1. Factor loadings of the latent slope were set to a model with an unspecified shape (ie, 0, 1, * and *) such that the third and fourth factor loadings of the slope could be freely estimated. Instead of assuming linear growth of each factor from the baseline to 9-month follow-up, it was more reasonable to have a model with an unspecified shape without such a strong assumption [44].

Second, conditional LGCM was conducted to examine the effects of the intervention on the outcome and potential mediators. Intervention condition (ie, mHealth intervention vs control) was explored as a predictor of changes in depressive symptoms, coping, and HIV-related stigma across time (ie, predicting the slope factor). A dummy variable was created to represent group assignment. The mHealth intervention group was coded as 1 and the control group as 0. A significant path from the intervention condition to the slope of the variable of interest would indicate a significantly larger change in that variable over time in intervention group than in control group.

Third, a longitudinal mediation model was used to investigate whether the mHealth intervention was effective in reducing depressive symptoms via the potential mediators by examining changes in the slopes of the outcome (ie, depressive symptoms) and mediators (ie, coping and HIV-related stigma). The mediating effects were estimated using bias-corrected 95% bootstrapped CIs (BCIs) with resampling of 5000 [45-47].

All LGCMs were conducted using maximum likelihood estimation. Model fit was assessed using the comparative fit index (CFI) and the root mean square error of approximation (RMSEA), the standardized root mean square residual (SRMR), and the relative chi-square ratio (chi-square/df). A LGCM model with a good model fit met the following criteria: CFI>0.90, RMSEA<0.08, SRMR<0.08, and relative chi-square ratio<3.0 [48,49]. Preliminary statistical analyses were performed using R software, version 3.5 (R foundation for Statistical Computing). LGCM analyses were performed using Mplus software, version 7 (Muthén & Muthén).

## Results

### Participant Enrollment

A total of 1555 PLWH were assessed for eligibility, among whom 1255 were excluded or withdrawn before enrollment. A total of 1017 were excluded because of lower CES-D scores (ie, CES-D<16), and 538 were further screened. Among the 538 participants, 164 declined to participate; 24 refused eligibility interview, and 50 were excluded because of other reasons such as currently taking psychotropic medication, participating in other studies, or unable to read because of eye problems. The RCT included 300 participants, with 150 in the intervention group and 150 in the control group. Details of the recruitment process are described in the study protocol [35]. Dropout rates for 3-, 6-, and 9-month follow-ups were 8.7% (26/300), 11.7% (35/300), and 13.3% (40/300), respectively. All demographic characteristics displayed in Table 1 were examined, and characteristics of dropouts were not statistically different from those who completed the study, aside from being slightly older. Reasons for dropping out included nonresponse (n=29), refusing to continue (n=9), transferring to another hospital (n=1), and imprisonment (n=1).

### Descriptive Analyses

**Figure 1:**
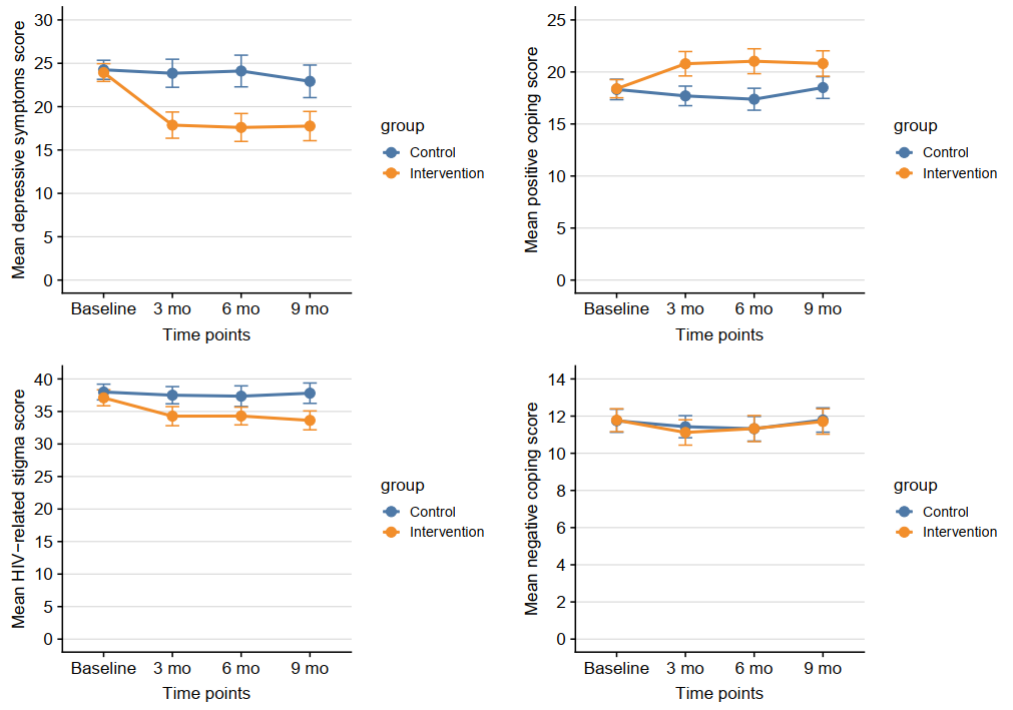
Measurements of depressive symptoms, coping, and HIV-related stigma over time. Error bars indicate 95% confidence intervals.

Descriptive statistics for baseline data are presented in Table 1 by group. There was no significant difference between the intervention and control groups except for sexual orientation, where more heterosexual participants were allocated in the control group. The participants were primarily male (277/300, 92.3%), employed (251/300, 83.7%), and with a median age (interquartile range) of 27.5 years (24.5-31.3). The majority (245/300, 81.7%) were homosexual or bisexual or uncertain of their sexual orientation. On average, participants in the intervention group completed 55% (33/60) of the 12 total Run4Love sessions at 3 months. Repeated measurements of the outcome variable (ie, depressive symptoms) and potential mediators (ie, HIV-related stigma and coping) at 4 time points for the intervention and control groups are shown in Table 2. The observed growth trajectories of mean depressive symptoms, coping, and HIV-related stigma scores for the intervention group and control group over 4 time points are presented in Figure 1. Our RCT indicated that the Run4Love mHealth intervention significantly reduced depressive symptoms and HIV-related stigma and improved positive coping compared with the control group at 3-, 6-, and 9-month follow-ups.

**Table 1:** Participants’ characteristics for the intervention and control groups at baseline.

Characteristics	Total (n=300)	Intervention (n=150)	Control (n=150)	*P* value
Age (years), median (interquartile range, IQR)	27.5 (24.5-31.3)	27.4 (24.3-31.1)	27.8 (24.6-32.2)	.40^a^
Male, n (%)	277 (92.3)	142 (94.7)	135 (90.0)	.19^b^
Educational level > high school, n (%)	182 (60.7)	98 (65.3)	84 (56.0)	.12^b^
Homosexual/bisexual/uncertain, n (%)	245 (81.7)	130 (86.7)	115 (76.7)	.04^b^
Married, n (%)	38 (12.7)	18(12.0)	20 (13.3)	.73^b^
Employed, n (%)	251 (83.7)	123 (82.0)	128 (85.3)	.53^b^
Family monthly income ≥7000 (yuan), n (%)	124 (41.3)	68 (45.3)	56 (37.3)	.20^b^
Duration since HIV diagnosis (years), median (IQR)	1.7 (0.6-3.7)	1.7 (0.6-4.0)	1.8 (0.6-3.9)	.62^c^
Center for Epidemiological Studies Depression Scale, mean (SD)	24.1 (6.6)	23.9 (6.4)	24.3 (6.9)	.68^a^
SWCQ^d^, positive coping, mean (SD)	18.4 (5.8)	18.4 (5.5)	18.3 (6.2)	.92^a^
SWCQ, negative coping, mean (SD)	11.8 (3.9)	11.8 (3.9)	11.8 (3.9)	.94^a^
HIV Stigma Scale, mean (SD)	37.5 (7.6)	37.1 (7.7)	38.0 (7.5)	.31^a^

^a^Based on *t* test.

^b^Based on chi-square test, the Fisher exact *P* values were used.

^c^Based on Wilcoxon rank-sum test.

^d^SWCQ: Simplified Ways of Coping Questionnaire.

**Table 2:** Repeated measurements of depressive symptoms and potential mediators in the Run4Love randomized controlled trial.

Variables, group		Baseline, mean (SD)	3-month follow-up, mean (SD)	6-month follow-up, mean (SD)	9-month follow-up, mean (SD)
**Depressive symptoms**					
	Intervention	23.93 (6.39)	17.87 (9.44)	17.60 (10.06)	17.86 (10.72)
	Control	24.25 (6.86)	23.85 (10.11)	24.11 (11.42)	23.43 (11.45)
**Positive coping**					
	Intervention	18.39 (5.45)	20.79 (7.33)	21.03 (7.48)	20.95 (7.75)
	Control	18.32 (6.15)	17.70 (5.88)	17.38 (6.59)	18.31 (6.41)
**Negative coping**					
	Intervention	11.78 (3.85)	11.12 (4.26)	11.33 (4.38)	11.71 (4.09)
	Control	11.75 (3.88)	11.43 (3.71)	11.32 (4.14)	11.87 (4.09)
**HIV-related stigma**					
	Intervention	37.10 (7.67)	34.28 (9.19)	34.30 (8.52)	33.98 (9.01)
	Control	37.99 (7.54)	37.50 (8.27)	37.35 (9.92)	37.79 (9.99)

### Latent Growth Curve Modeling

#### Intervention Effects

**Figure 2:**
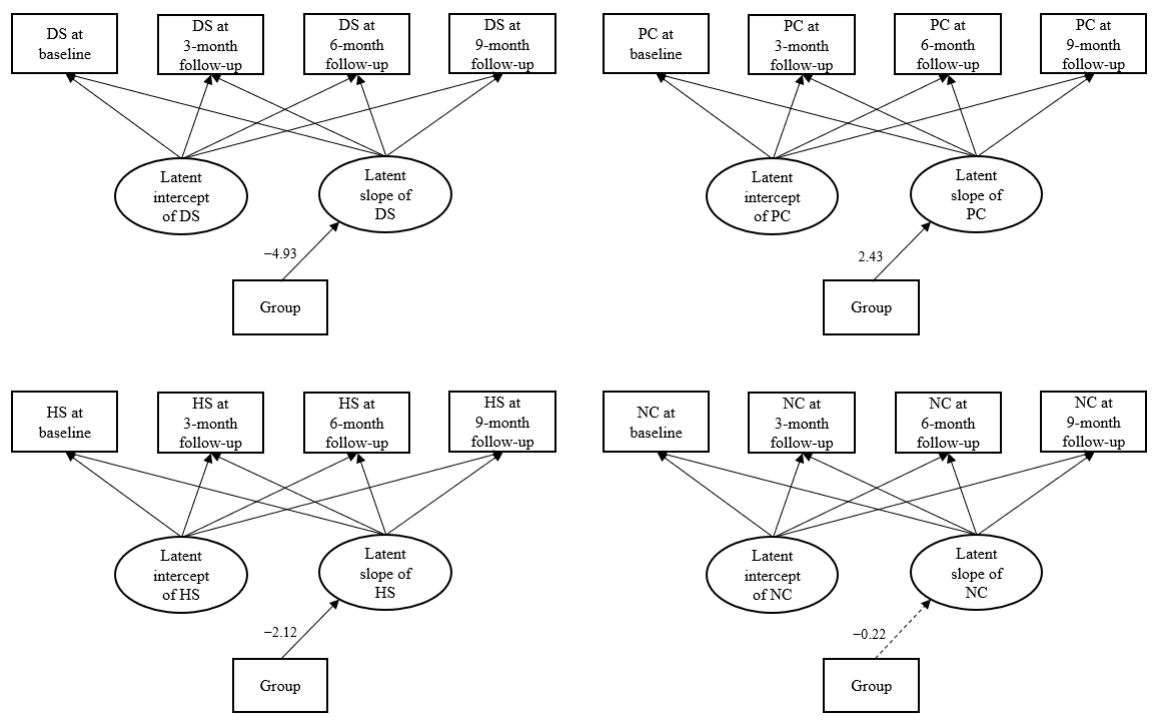
Conditional latent growth curve modeling examining the effects of the mobile health intervention on the outcome and potential mediators. Continuous lines with arrows indicate statistically significant paths. Dotted lines indicate nonsignificant paths. The first and second factor loadings of the latent slope of all models were set to 1, the third and fourth factor loadings of the latent slope of all models were freely estimated. Group: intervention or control group; DS: depressive symptoms; HS: HIV-related stigma; PC: positive coping; NC: negative coping.

Conditional LGCM supported the beneficial effects of the intervention on the outcome and potential mediators. Figure 2 presents the path diagram of each conditional LGCM. There were significant effects of the intervention on the slopes of depressive symptoms (beta=–4.93; *P*<.001), positive coping (beta=2.43; *P*<.001), and HIV-related stigma (beta=–2.12; *P*<.001). The results indicated that there was significantly more reduction in depressive symptoms and HIV-related stigma and significantly more improvement in positive coping over time in the intervention group than in the control group. The LGCMs for the abovementioned 3 variables showed good model fit. Table 3 presents model fit indices for all LGCMs. In addition, the intervention did not have a significant intervention effect on the slope of negative coping (beta=–0.22; *P*=.49), indicating that there was no significant reduction in negative coping over time in the intervention group compared with the control group. Therefore, negative coping was not included in the final analyses of mediating effects.

**Table 3:** Model fit indices of all latent growth curve models.

Model	CFI^a^	RMSEA^b^	SRMR^c^	Relative chi-square ratio (df)
Reference	>0.90	<0.08	<0.08	<3.0
LGCM^d^ for depressive symptoms	1.00	0.04	0.02	1.4 (7)
LGCM for positive coping	1.00	0.00	0.03	0.8 (7)
LGCM for negative coping	1.00	0.02	0.04	1.1 (7)
LGCM for HIV-related stigma	1.00	0.00	0.02	0.8 (7)
Final LGCM	0.98	0.05	0.04	1.7 (56)

^a^CFI: comparative fit index.

^b^RMSEA: root mean square error of approximation.

^c^SRMR: standardized root mean square residual.

^d^LGCM: latent growth curve model.

#### Mediating Effects of Positive Coping and HIV-Related Stigma

**Figure 3:**
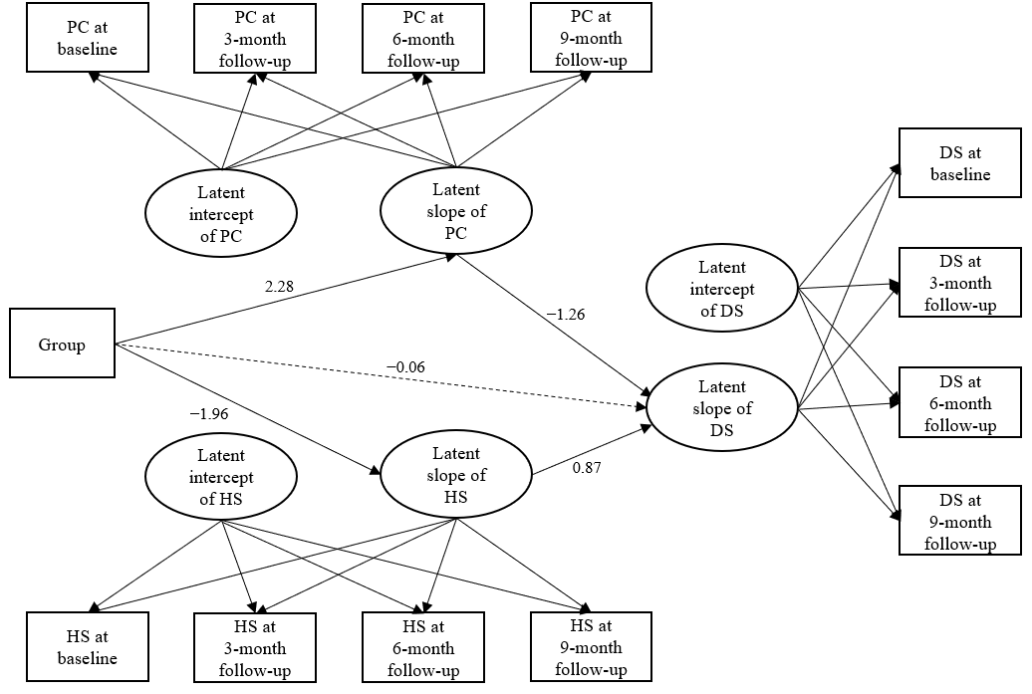
Latent growth curve modeling examining mediating effects between the mobile health intervention and changes in depressive symptoms. Continuous lines with arrows indicate statistically significant paths. Dotted lines indicate nonsignificant paths. The first and second factor loadings of the latent slope of the model were set to 1, the third and fourth factor loadings of the latent slope of the model were freely estimated. Group: intervention or control group; PC: positive coping; HS: HIV-related stigma; DS: depressive symptoms.

The path diagram of LGCM in Figure 3 shows how the mHealth intervention reduced depressive symptoms via mediators of positive coping and HIV-related stigma. Pathways and BCIs of the final parallel-process LGCM are presented in Table 4. The final model indicated good model fit (CFI=0.98, RMSEA=0.05, SRMR=0.04, relative chi-square ratio (df)=1.7 (56). The results of the parallel-process LGCM indicated significant mediating effects of positive coping and HIV-related stigma on depression reduction in the mHealth intervention.

The results indicated a significantly indirect effect of the intervention on the slope of depressive symptoms via the slope of positive coping (beta=2.275×(−1.257)=−2.86; 95% BCI −4.78 to −0.94). This means that the mHealth intervention significantly improved participants’ positive coping over time, which in turn significantly reduced depressive symptoms of the participants over time. Similarly, there was also a significantly indirect effect of the intervention on the slope of depressive symptoms via the slope of HIV-related stigma (beta=−1.962)×0.873=−1.71; 95% BCI −3.03 to −0.40), indicating significant intervention effects in reducing HIV-related stigma, which in turn significantly reduced depressive symptoms of the participants over time.

The direct effect of the intervention on the slope of depressive symptoms was not statistically significant (beta=−0.06; 95% BCI −2.15 to 2.03) when the mediators were added, indicating no direct effect of the intervention on depressive symptoms of the participants. Therefore, the effects of the mHealth intervention Run4Love in reducing depressive symptoms of the participants might be largely explained by the indirect effects of the intervention on enhancing positive coping and reducing HIV-related stigma among PLWH.

**Table 4:** Coefficients and bootstrapping CIs of the final parallel-process latent growth curve modeling.

Effect		Estimate	95% BCI^a^	Standardized estimate
**Direct effect on the slope of depressive symptoms**				
	Slope of positive coping	−1.26^b^	−1.89 to −0.62	−0.59
	Slope of HIV-related stigma	0.87^b^	0.48 to 1.27	0.52
	Group	−0.06	−2.15 to 2.03	−0.00
	Intercept of positive coping	−0.22	−0.47 to 0.04	−0.15
	Intercept of HIV-related stigma	0.08	−0.06 to 0.22	0.08
**Direct effect on the slope of positive coping**				
	Group	2.28^b^	1.06 to 3.50	0.37
	Intercept of depressive symptoms	0.20^b^	0.04 to 0.36	0.37
	Intercept of HIV-related stigma	−0.10	−0.22 to 0.02	−0.21
**Direct effect on the slope of HIV-related stigma**				
	Group	−1.96^b^	−3.22 to−0.70	−0.25
	Intercept of positive coping	−0.35^b^	−0.57 to−0.13	−0.40
	Intercept of depressive symptoms	−0.14^b^	−0.28 to−0.01	−0.21
**Indirect effect of group on the slope of depressive symptoms**				
	Via the slope of positive coping	−2.86^b^	−4.78 to−0.94	−0.22
	Via the slope of HIV-related stigma	−1.71^b^	−3.03 to−0.40	−0.13
Total indirect effect		−4.57^b^	−7.01 to −2.14	−0.35

^a^BCI: bootstrapped CIs.

^b^CI does not contain zero.

## Discussion

### Principal Findings

Our study is among the first to examine mediating factors of the effects of an mHealth intervention designed for depression reduction among PLWH. In addition, this study is the first study that has utilized latent growth curve modeling for examining mediators in an mHealth intervention study using 4 time-point measurement data among PLWH. We found that enhancement of positive coping and reduction of HIV-related stigma were important mediating factors of the mHealth intervention in reducing depression among PLWH.

Previous studies using qualitative analysis have demonstrated a mediating effect of positive coping on achieving depression reduction in face-to-face interventions [25,50]. Results of the LGCM in our quantitative analysis affirmed this finding in the Run4Love mHealth intervention [25,50]. This finding underscored the critical role of positive coping in depression reduction and suggested that enhancing participants’ positive coping may be an effective treatment strategy to reduce depression among PLWH in mHealth-based interventions.

One reason for the significant enhancement of positive coping among PLWH might be that the Run4Love mHealth intervention was adapted from evidence-based CBSM program, with an important component of training in coping skills [21]. In the intervention, participants were instructed to practice positive coping skills by listening to audio recordings and reading short essays delivered via our enhanced mHealth platform [35]. The short essays and audio recordings on effective coping skills included problem- and emotion-focused coping and relaxation exercises. As reported in the previous studies of face-to-face CBSM interventions, problem- and emotion-focused coping and relaxation exercises were effective ways of improving positive coping among PLWH [51,52].

One advantage of the Run4Love mHealth intervention over traditional face-to-face interventions may be that the materials (eg, audio clips and essays) can be repeated, read, or heard at any time or location of participants’ choice. The tracking and monitoring functions of the Run4Love intervention can provide timely feedback to both researchers and participants, such as whether and for how long each participant read or listened to the materials sent via the mHealth platform. Instead of recalling what is learned in face-to-face sessions or seeking clinicians’ suggestions in traditional interventions, participants in mHealth interventions are able to read, listen to, and review materials related to positive coping skills whenever they encounter challenges in their daily lives [53,54]. Therefore, as opposed to traditional interventions, mHealth interventions such as Run4Love have the advantage of delivering psychological materials and support with increased accessibility, lower cost, and increased privacy for participants. These characteristics, in turn, increase the potential to scale-up the intervention for widespread implementation, especially in resource-limited settings [28].

The results of this study also revealed that depression reduction in the Run4Love mHealth intervention was significantly mediated by reduction of HIV-related stigma. Previous studies found that face-to-face cognitive behavioral interventions were effective in reducing HIV-related stigma and depression in PLWH [25,55,56]. For example, 1 study showed that a face-to-face cognitive behavioral intervention was effective in reducing stigma, which in turn led to alleviated emotional symptoms in outpatients with anxiety and depressive symptoms in a pre- and postintervention assessment design [57]. Our study contributed to existing literature by demonstrating that an mHealth intervention can significantly reduce HIV-related stigma and also that reduced HIV-related stigma served as an important mediator in the overall effect of the mHealth intervention on depression reduction.

Although the specific features of the Run4Love intervention that might explain the significant reduction in HIV-related stigma are not clear, 2 possible elements might be (1) the important component of HIV stigma reduction messages in the CBSM and (2) the additional 5 phone calls from research staff that occurred throughout the study. The phone calls may have served as additional social support for the participants as research staff helped to facilitate participation, improve intervention adherence, and provide feedback and guidance on participants’ mental health status. Previous research indicates that social support is an important factor in reducing HIV-related stigma [58]. Our study findings provided empirical evidence that the Run4Love mHealth intervention decreased the experience of HIV-related stigma, which in turn effectively reduced depressive symptoms among PLWH.

### Limitations

There were several limitations in this study. First, although our study focused on positive coping and HIV-related stigma, other factors such as stress, self-efficacy, physical activity, patient satisfaction, guidance (working alliance), emotion regulation skills, and expectations might also serve as potential mediating factors for depression reduction among PLWH in the Run4Love mHealth intervention. Social support that the research staff may have provided with the phone calls might also serve as potential mediators, but we did not measure social support in this study. Future studies should further assess and explore the potential mediating effects of these factors in mHealth interventions. Second, data in this study were self-reported, which might introduce recall and social desirability biases. More objective measures such as biomarkers could be incorporated in the future studies. Third, as the data in this study were collected in an urban setting, caution should be exercised when generalizing the results to other places such as rural areas. Fourth, as the temporal sequence could not be identified between the mediators and outcome in this study, a causal relationship between the mediators and outcome cannot be confirmed. Fifth, selection bias in enrollment may limit the generalizability of the findings. Sixth, mediators only statistically narrow the mechanisms of change and might not necessarily be congruent [12,13]. Despite these limitations, this study provided additional empirical and quantitative evidence for a better understanding of the effects of an mHealth intervention on depression reduction among PLWH through mediating factors.

### Conclusions

In conclusion, this study revealed several mediating effects of an mHealth intervention using latent growth curve modeling and 4 time-point longitudinal measurement data. Positive coping and HIV-related stigma were important mediating factors of the Run4Love mHealth intervention in reducing depression among PLWH. The study’s findings provided empirical evidence for future research to enhance positive coping and reduce HIV-related stigma in mHealth interventions to reduce depression among PLWH. In addition, future interventions and policies aimed at reducing depression among PLWH should be designed with specific features that address positive coping and stigma to maximize intervention efficacy.

## Appendix

Multimedia Appendix 1 CONSORT-EHEALTH checklist (V 1.6.1).

## Abbreviations

BCI: bootstrapped CI
CBSM: cognitive behavioral stress management
CES-D: Center for Epidemiological Studies Depression Scale
CFI: comparative fit index
LGCM: latent growth curve model
mHealth: mobile health
PLWH: people living with HIV
RCT: randomized controlled trial
RMSEA: root mean square error of approximation
SEM: structural equation modeling
SRMR: standardized root mean square residual
SWCQ: Simplified Ways of Coping Questionnaire
